# Bone mineral density in Palestinian patients with end-stage renal disease and the related clinical and biochemical factors: Cross-sectional study

**DOI:** 10.1371/journal.pone.0241201

**Published:** 2020-11-12

**Authors:** Zaher Nazzal, Shahd Khader, Hiba Zawyani, Mazen Abdallah, Osama Sawalmeh, Zakaria Hamdan

**Affiliations:** 1 Family and Community Medicine Department, Faculty of Medicine and Health Sciences, An-Najah National University, Nablus, Palestine; 2 Medicine Department, Faculty of Medicine and Health Sciences, An-Najah National University, Nablus, Palestine; 3 Orthopedic Surgery Department, An-Najah National University Hospital, An-Najah National University, Nablus, Palestine; 4 Internal Medicine Department, An-Najah National University Hospital, An-Najah National University, Nablus, Palestine; 5 Nephrology Consultant, Nephrology Department, An-Najah National University Hospital, An-Najah National University, Nablus, Palestine; Charles P. Darby Children's Research Institute, 173 Ashley Avenue, Charleston, SC 29425, USA, UNITED STATES

## Abstract

**Introduction:**

End-Stage Renal Disease (ESRD) is the ultimate result of chronic kidney disease (CKD). In Palestine, the prevalence of ESRD was 240.3 PMP which is comparable with the nearby countries. Accelerated bone loss among ESRD patients is attributed to abnormal bone turn over that leads to osteoporosis and osteopenia. The risk of fractures is increased four-fold in men and women on hemodialysis, which explains the importance of assessing the bone mineral density among these population. The goals of this study were to find the prevalence of osteoporosis in ESRD patients as determined by bone mineral density (BMD) at different sites and to determine whether BMD correlates with many other clinical parameters.

**Methods:**

A cross-sectional study of 194 ESRD patients were recruited from the dialysis unit in An-Najah National University Hospital, Nablus, Palestine. The patients were on regular hemodialysis or peritoneal dialysis. BMD was measured at the lumbar spine and the hip using the dual-energy X-Ray absorptiometry (DEXA) and the value is expressed as T-score. The data were analyzed using SPSS, version 26. The relationship between BMD and the clinical and biochemical parameters among the ESRD patients was assessed.

**Results:**

We found that 42.8% of ESRD patient had osteoporosis and 40.2% had osteopenia. There were significantly higher proportions of osteoporosis and osteopenia among patients >60 years of age (p<0.005). Patients with osteoporosis and osteopenia had significantly higher serum levels of PTH (792.9 and 469.7) (p<0.05). BMD decreases as the duration of dialysis (39.0 months Vs. 56.8 months), (p<0.05). We found no significant difference between patients on hemodialysis or peritoneal dialysis.

**Conclusion:**

This study showed that Palestinian patients with ESRD have low BMD at the hip and spine. The observed high serum level of PTH was associated with low BMD. Those patients should be closely monitored especially those with more than one risk factor. Moreover, more attention should be paid for these category of patients to decrease the incidence of falling down and the resulting fractures that might lead to mortality and morbidity.

## Introduction

End-Stage Renal Disease (ESRD) is the ultimate result of chronic kidney disease (CKD) [1]. CKD is defined as decreased kidney function, as shown by glomerular filtration rate (GFR) of less than 60 mL/min per 1.73 m^2^, or markers of kidney damage, or both, for at least 3 months, regardless of the underlying cause [2]. The normal level of GFR varies by age, sex and body size with the normal GFR in young adults is considered approximately 120 to 130 ml/min/1.73 m^2^ and it normally declines with age [3].

According to KDOQI- Kidney Disease Outcomes Quality Initiative- guidelines [4], CKD stage 5 or end ESRD is defined as: GFR less than 15 ml/min/1.73 m^2^, which is accompanied in most cases by signs and symptoms of uremia and, eventually, the need for kidney replacement therapy, dialysis or transplantation [2, 3–5].

In 2018, the measured ESRD prevalence in the Middle East is estimated to be 360 per million population (PMP) [6] compared with 2160 PMP in the US in 2016 [7]. In Palestine, the prevalence of ESRD was 240.3 PMP [8] which is comparable with the nearby countries.

Accelerated bone loss among ESRD patients is attributed to abnormal bone turn over that leads to the status of bone problems, osteoporosis and osteopenia [9].

Secondary hyperparathyroidism is a common complication of CKD and it is attributed to hyperphosphatemia, hypocalcemia, reduced vitamin D synthesis as well as PTH skeletal resistance [5, 10, 11]. The excessive secretion of PTH among renal failure patients results in increased bone turnover [11–13]. In addition to high PTH levels, other risk factors are linked to low bone density such as advanced age, low serum albumin level as well as high serum alkaline phosphatase levels [9, 10].

To examine the strength of the bones, there are qualitative (Bone Biopsy) and quantitative (densitometry) methods. DEXA scan is recognized as the best available method worldwide to assess bone density because of its high accuracy, short scan time and low radiation dose [9, 14].

The results of DEXA scans can be expressed as BMD (g/cm2), Z-score or T-score. The T-score is the number of standard deviations (SD) from the mean of a healthy young adult, while Z-score is the number of SD from the mean of healthy age and gender-matched populations [15].

DEXA scans can be performed at different parts of the body with the most common skeletal parts to be measured are lumbar spine (LS) and proximal femur [15]. The International Society for Clinical Densitometry (ISCD) recommends BMD measurement of the postero-anterior LS (L1–L4) and hip (total and neck) [16].

Prevalence of bone disorders, including osteoporosis, in hemodialysis patients is estimated to be between 33–66% [14] The tendency to fracture is inversely correlated with BMD, confirming the importance of BMD measurement [17]. According to the WHO, The diagnosis of osteoporosis is applied when the T score on Bone-Mineral Density measurement using DEXA scan is -2.5 or lower [15, 18]. When the T score is between -1.0 and -2.5 the term is referred to as Osteopenia [19]. It has been demonstrated that the risk of fractures is increased four-fold in men and women on hemodialysis [5, 20].

Other studies have shown that the yearly incidence rate for fractures by site is approximately 1% for hip fractures and 2.5% for any fracture affecting ESRD patients [5]. Following a hip fracture, patients with ESRD have a 1-year mortality rate twice that of other hemodialysis patients [20].

The aim of this study is to assess the level of bone mass density in the hip and Lumbar Spine of patients undergoing dialysis and to find its correlation with some clinical and biochemical factors

## Materials and methods

### Study design, setting and population

This cross-sectional correlation study was conducted between November 2018 and February 2019 at the dialysis center of An-Najah National University Hospital (NNUH), Nablus, Palestine. This unit is the largest dialysis center in the region with more than 350 patients receiving hemodialysis and peritoneal dialysis therapy.

All participants were ESRD patients on regular hemodialysis (three times weekly, 4 hours per session) or peritoneal dialysis (Continuous ambulatory peritoneal dialysis) from both genders. We excluded children and patients with a history of bone malignancy.

All participants were provided oral and written informed consents for participation in the study. The study was carried out with the approval of The Institutional Review Board of An-Najah National University. The principal investigator invited the eligible subjects to participate in the study and 194 of them approved their participation.

### Date collection

Clinical and demographic characteristics were collected from the participants and their medical records. These included age, gender, number of years on dialysis, diabetic status (yes, no), Hypertension status (yes, no), Type of dialysis (Hemodialysis or Peritoneal dialysis), history of transplantation (yes, no), history of fractures (yes, no) in addition to the relevant medication history (Alfacalcidol, calcium supplements and phosphate binders, sevelamer). The biochemical parameters were: serum levels of albumin, calcium, phosphate, alkaline phosphatase (ALP) and parathyroid hormone (PTH). The biochemical measurements were conducted on a monthly basis with the exception of PTH which was measured every three months. All biochemical parameters were collected before the start of the dialysis session on all data collection occasions. All blood samples were sent to the laboratory for analysis immediately after collection and were analyzed on the same day. One set of blood sample was analyzed for every single patient. The measurement procedures and reports were validated by the Division of Biochemistry and Laboratory at NNUH.

### Bone Mineral Density (BMD)

BMD was measured using dual-energy X-Ray absorptiometry (DEXA) scan (Hologicapparatus model Discovery WI S/N 82189). It was performed within the same month of all biomedical parameters by trained technicians at Rahma Medical center, Nablus. We measured BMD at the LS and the hip. The LS was measured in posterior–anterior (PA at L1–L4) projection. The hip was measured in the area of the neck, the trochanter (Troch), the intertrochanteric (Inter), the total hip, and Ward’s Triangle. The results are expressed as BMD (g/cm2), a T-score.

### Statistical analyses

The biochemical and demographic characteristics of the participants were summarized using the descriptive statistics. Means with standard deviation (SD) were used to summarize continuous variables and frequencies with percentages for categorical variables. We used ANOVA, Kruskal Wallis test, independent *t*-test and Chi-squared test to examine for any statistically significant differences between the different groups as appropriate. All outcome variables were normally distributed and no data transformation was needed. Ordinal logistic regression was used to control for gender, age, duration of dialysis and variables found to be significant in univariable analysis. Any p-value less than 0.05 is considered to be statistically significant and all analyses were conducted using the SPSS computer software version 26.0 (IBM Corp).

## Results

### Baseline characteristics of the patients

One hundred ninety four patients were enrolled and all of them completed the study. One hundred eighty four were on hemodialysis and the remaining ten patients were on peritoneal dialysis.

The mean age of participants was 57.0 years (SD = 14.5) and 114 patients (58.8%) were males. About 52.1% (n = 101) and 78.4% (n = 152) had diabetes and hypertension, respectively. The mean duration on hemodialysis was 48 months. Only sixteen patients (8.2%) had tried renal transplantation and 17.5% of the patients had experienced a prior fracture. The baseline demographic, biochemical and clinical characteristics of patients are summarized in Table 1.

**Table 1:** *Baseline* clinical and biochemical characteristics of the participants (194).

*Variables*	*Frequency (%)*	Mean±SD (Range)
**Age** (years)		57.0± 14.5 (18.0–88.0)
<60	102 (52.6%)	
≥60	92 (47.4%)	
***Gender***		
Male	114(58.8%)	
Female	80(41.2%)	
***Duration of Dialysis*** (months)		48.0±45.0 (2.0–228.0)
***Type of Dialysis***		
Hemodialysis	184 (94.8%)	
Peritoneal Dialysis	10 (5.2%)	
***History of renal transplantation*** (yes)	16 (8.2%)	
***History of fractures*** (yes)	34 (17.5%)	
***Diabetes Mellitus*** (yes)	101 (52.1%)	
***Hypertension*** (yes)	152 (78.4%)	
***Alpha D3 Supply*** (yes)	185(92.5%)	
***Calcium Supplemtation*** (yes)	169(87.1%)	
***Sevelamer Supplementation*** (yes)	24 (12.0%)	
***Calcium Supplemtation dose*** (mg/day)		923.0±358.9 (177.4–2155)
***Alpha D3 Supplemtation dose*** (mcg/day)		0.700± 0.48 (0.25–2.75)
***Sevelamer Supplemtation dose*** (mg/day)		2566.3±1112 (700–4200)
***Serum Calcium level*** (mg/dl)*		8.8±0.95 (3.9–11.5)
***Serum PTH level*** (pg/ml)		605.5±772.9 (25.9–6244.0)
***Serum Phosphorus level*** (mg/dl)		4.75±1.43 (1.7–11.1)
***Serum Albumin level*** (g/dl)		3.8±0.34 (2.7–4.6)

*Calcium is not corrected with albumin

### Bone densinometric data (BMD)

**Fig 1:**
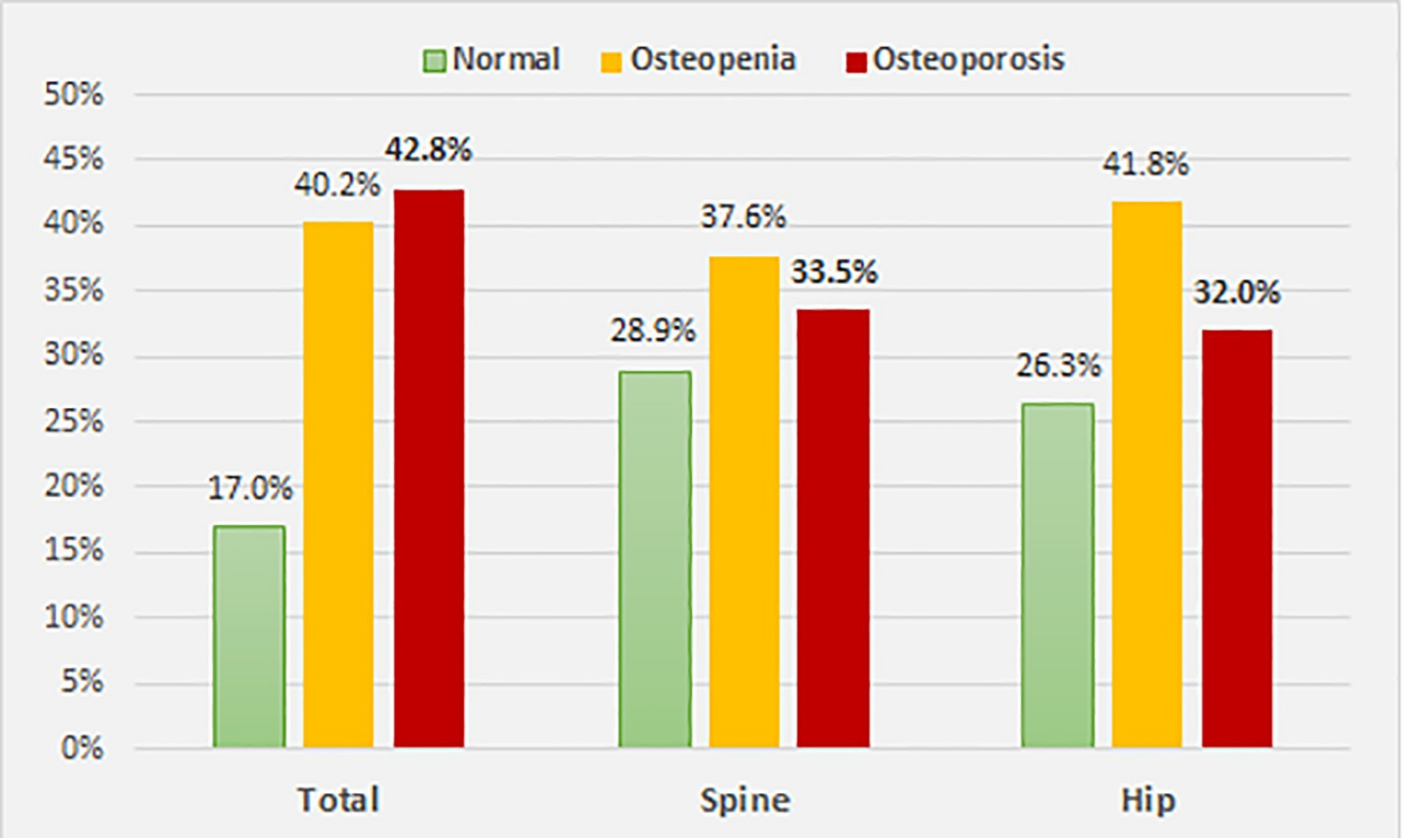
Distribution of bone mass density among participants (n = 194).

The overall prevalence of osteoporosis among the participants was 42.8% whilst the proportion of people who had osteopenia was 40.2%. According to the site, 33.5% of patients had osteoporosis in the LS and 32.0% in the Hip. *See* Fig 1.

### BMD in relation to laboratory and clinical parameters

Univariable analysis was conducted to assess the relationship between patients' BMD status, and their background and clinical characteristics. Proportions of osteopenia and osteoporosis were significantly higher among patients ≥60 years of age relative to patients <60 years of age for LS and Hip BMD (p<0.05). For gender, females have significantly had higher LS osteoporosis compared to males (P = 0.033).

There was a significant relationship between BMD and PTH levels. Osteopenia and osteoporotic patients had higher serum levels of PTH, this was statistically significant for the Total BMD (P = 0.008) and Hip BMD (P = 0.019). This relation remains significant after controlling for gender, age and duration of dialysis. See Table 2. For the Hip BMD, Post hoc analysis showed that PTH is significantly higher among osteoporosis group compared to osteopenia group (P = 0.006) and compared to normal group (P = 0.034). For total BMD, the significant difference was observed between osteoporosis group compared to osteopenia group (P = 0.022). Serum calcium was on average higher among the osteoporotic and osteopenic patients in all BMD studies and the difference was statistically significant among the spine BMD (P<0.05). Post hoc analysis showed that this difference is mainly significant between the normal and osteopenia group (P = 0.041).

**Table 2:** Univariable and multivariable comparison of clinical and biological characteristics with BMD status.

Parameters		Normal (n = 33) †	Osteopenia (n = 78) †	Osteoporosis (n = 83) †	P value	Adjusted P value**
**Age**						
<60	Total	21 (20.6%)	46 (45.1%)	35 (34.3%)	0.128	0. 080
	Lumbar spine	30 (29.4%)	44 (43.1%)	28 (27.5%)	0.046	0.399
	Hip	32 (31.4%)	45 (44.1%)	25 (24.5%)	0.039	0.002
≥60	Total	12 (13.0%)	32 (34.2%)	48 (52.2%)	0.128	0.080
	Lumbar spine	26 (28.3%)	29 (31.5%)	37 (40.2%)	0.046	0.399
	Hip	19 (20.7%)	36 (39.1%)	37 (40.2%)	0.039	0.002
**Gender**						
*Male*	Total	22(19.3%)	46(40.4%)	46 (40.4%)	0.539^#^	0.678
	Lumbar spine	41(36.0%)	38 (33.3%)	35 (30.7%)	0.033^#^	0.173
	Hip	31 (27.2%)	51 (44.7%)	32 (28.1%)	0.371^#^	0.752
*Female*	Total	11(13.8%)	32(40.0%)	37(46.3%)	0.539^#^	0.678
	Lumbar Spine	15(18.8%)	36 (43.8%)	30 (37.5%)	0.033^#^	0.173
	Hip	20 (25.0%)	30 (37.5%)	30 (37.5%)	0.371^#^	0.752
**Dialysis Type**						
*Hemodialysis*	Total	32(17.4%)	75 (40.8%)	77 (41.8%)	0.520^#^	---
	Lumbar Spine	54(29.3%)	70(38.0%)	60(32.6%)	0.520^#^	---
	Hip	48(26.1%)	78(42.4%)	58(31.5%)	0.743^#^	---
*Peritoneal Dialysis*	Total	1(10.0%)	3(30.0%)	6(60.0%)	0.520^#^	---
	Lumbar Spine	2(20.0%)	3(30.0%)	5(50.0%)	0.520^#^	---
	Hip	3(30.0%)	3(30.0%)	4(40.0%)	0.571^#^	---
**Duration on Dialysis (***Months***)**						
	Total	39.0±39.8	41.3±41.2	57.8±48.8	0.030*	0.021
	Lumbar Spine	34.8±36.0	51.1±47.3	55.8±46.8	0.028*	0.050
	Hip	38.7±39.6	44.3±43.7	59.8±48.1	0.012*	0.005
**History of Transplantation (***Yes***)**						
**Yes**	Total	4(25.0%)	4(25.5%)	8(50.0%)	0.393^#^	---
	Lumbar Spine	5(31.3%)	2(12.5%)	9(56.3%)	0.059^#^	---
	Hip	3(18.8%)	8(50.0%)	5 (31.3%)	0.718^#^	---
**History of Fractures**						
**Yes**	Total	3(8.8%)	11(32.4%)	20(58.8%)	0.094^#^	---
	Lumbar Spine	6(17.6%)	12(35.3%)	16(47.1%)	0.128^#^	---
	Hip	6(17.6%)	12(35.3%)	16(47.1%)	0.104^#^	---
**Diabetes Millitus (***Yes***)**						
	Total	16(15.8%)	46(45.4%)	39(38.6%)	0.284^#^	---
	Lumbar Spine	32(32.7%)	39(38.6%)	30(29.7%)	0.462^#^	---
	Hip	25(24.8%)	44(43.6%)	32(31.7%)	0.538^#^	---
**Hypertension (***Yes***)**						
	Total	26(17.1%)	63(41.4%)	63(41.4%)	0.754^#^	---
	Lumbar Spine	46(30.3%)	58(38.2%)	48(31.6%)	0.521^#^	---
	Hip	29(17.0)	63(41.2)	64(41.8)	0.534^#^	---
**Serum Calcium**						
	Total	8.7±1.40	8.8±0.84	8.9±0.81	0.053*	0.092
	Lumbar Spine	8.5±1.2	9.1±0.8	8.7±0.8	0.004*	0.921
	Hip	8.7±1.2	8.8±0.8	8.9±0.8	0.568*	---
**Serum PTH**						
	Total	454.8±378.9	469.7±478.3	792.9±1018.7	0.008**	0.012
	Lumbar Spine	448.9±402	592.4±613	766.2±1113	0.083**	0.554
	Hip	507.3±391	465±465	869.2±1186	0.019**	0.014
**Serum Phosphorus**						
	Total	5.0±1.43	4.7±1.27	4.7±1.65	0.481*	---
	Lumbar Spine	4.9±1.4	4.7±1.2	4.7±1.7	0.621*	---
	Hip	5.1±1.5	4.7±1.4	4.6±1.3	0.126*	---
**Serum Albumin**						
	Total	3.9±0.28	3.9±0.35	3.7±0.33	0.401*	---
	Lumbar Spine	3.9±0.3	3.9±0.4	3.7±0.3	0.013*	0.025
	Hip	3.9±0.32	3.8±0.33	3.7±0.3	0.019*	0.010
**Elemental Calcium supplement dose**						
	Total	1001±354	939.9±370	872.3±347	0.620*	---
	Lumbar Spine	1055±373	906±369	830±328	0.005*	0.201
	Hip	939±346	960±380	858±339	0.275*	---
**Alfacalcidiol dose**						
	Total	0.68±0.37	0.70±0.48	0.70±0.51	0.387*	---
	Lumbar Spine	0.69±0.35	0.72±0.50	0.69±0.50	0.940*	---
	Hip	0.66±0.39	0.70±0.49	0.72±0.51	0.750*	---
**Phosphorus binders dose**						
	Total	2880±1073	2171±390	3200±1563	0.064*	---
	Lumbar Spine	2628±1002	2666±1093	3054±155	0.751*	---
	Hip	2800±979	2755±1702	2933±1058	0.958*	---

*ANOVA test ** Kruskal Wallis Test

# Chi-square test

†This represent the total numbers

**Adjusted for age, gender, duration of dialysis.

Duration of dialysis was inversely associated BMD. Total, LS and hip osteoporotic patients had significantly higher duration of dialysis compared to osteopenia and normal patients (p = <0.05). This relation remains significant after controlling for gender, age and duration of dialysis, see Table 2. Post hoc analysis showed that duration of dialysis was significantly higher among total osteoporotic patients compared to osteopenia patients (P = 0.04), hip osteoporotic patients compared to normal patients (P = 0.013), and LS osteoporotic patients compared to normal patients (P = 0.028). Serum albumin was lower among osteoporotic patients according to Spine and Hip BMD (P<0.05). On post hoc test, hip osteoporotic patients had significantly lower level of albumins compared to osteopenia group (P = 0.041) and compared to normal group (P = 0.021). For LS BMD, the difference in serum albumin level was significant between osteoporosis and normal groups (P = 0.02). Moreover, in the three occasions, osteoporotic patients used to ingest less calcium supplements than the osteopenia and the normal patients (P<0.05 in the Spine BMD).

There were no significant differences in BMD between patients on HD and PD although the percentage of HD patients with normal BMD is higher than in PD patients. Even though 50% of patients with renal transplantation had osteoporosis, the association between BMD and renal transplantation was not significant.

According to Spearman correlation, there was a significant negative correlation (p<0.05) between duration of dialysis and both LS BMD and hip BMD. BMD (t-score) decreases as the duration of dialysis increases and this increases the risk of osteoporosis. PTH was also negatively correlated with LS and hip BMD (p<0.01, p<0.05 respectively). There was no correlation between hip BMD and either blood albumin level or doses of calcium carbonate, but both variables were correlated with LS BMD. We found a significant positive correlation between age and LS BMD, but not with hip BMD. Furthermore, there was no statistically significant correlation between hip and LS BMD with blood calcium or phosphorus level (Table 3).

**Table 3:** Spearman correlation of Spinal and Hip BMD with biochemical measurements.

Characteristic	Spine BMD r (p-value)	Hip BMD r (p-value)
**Age**	0.057 (0.412**)	-0.121 (0.093)
**Duration of dialysis**	-0.199 (0.005*)	-0.195 (0.006*)
**Serum Calcium Level**	0.024 (0.736)	0.039 (0.736)
**Serum PTH Level**	-0.201 (0.005**)	-0.195 (0.012*)
**Serum Phosphorous level**	0.07 (0.341)	0.142 (0.48)
**Serum Albumin Level**	0.24 (0.001**)	0.14 (0.089)
**CA Carbonate**	0.21 (0.009*)	0.096 (0.236)
**Ca Acetate**	0.08 (0.761)	-0.03 (0.923)
**AlphaD3**	-0.03 (0.758)	0.02 (0.822)
**Sevelamer**	0.584	0.794
**Calcium supplementation dose**	0.191(0.016*)	0.073 (0.365)

*Correlation is significant at 0.05 (2-tailed)

** Correlation is significant at 0.01 (2-tailed)

## Discussion

The aim of this cross-sectional study was to assess the prevalence of BMD among ESRD patients who undergo regular dialysis and the relation with other clinical and biochemical factors.

This study showed that most of ESRD patients had significantly low BMD, including osteopenia and osteoporosis, with a percentage of 83%, 71.1% and 71.1% at the total, hip and spine, respectively. See Fig 1. This is close to the findings of previous studies [14, 15, 21].

Low BMD was found to be associated with increased total duration of dialysis, higher serum PTH level, higher serum calcium level and decreased intake of elemental calcium supplement which confirms the findings of previous studies [9, 14, 22–24].

The percentage of female with osteoporosis in our study was higher than men in the three sites but the difference was statistically significant in the spine BMD. This trend is similar to previous studies [15, 24]. Zayour et al reported a significantly higher prevalence of osteoporosis among women (55% in men and 87% in women undergoing HD); and suggested that sex, diabetes mellitus, and duration of HD were predictors of low BMD [21]. We attribute this difference to the lower population of this study (28 Vs. 194) and the difference among gender distribution (20 males vs. 8 females).

This study showed no significant difference in BMD between patients on HD and PD (p = 0.571), which is in line with the findings of Nybo M et al. [25].

Higher PTH level is an indicator of lower BMD as patients with lower total BMD have higher serum levels of PTH, 792.9 pg/mL among osteoporotic patients compared with 469 pg/mL and 454 pg/mL among osteopenic and normal patient, respectively. See Table 2. This is similar to the findings of a meta-analysis that found serum PTH levels were negatively correlated or, at least, not related with bone density but none found a positive association [26]. Our study data revealed a significantly negative correlation between PTH levels and BMD in Total BMD and Hip BMD which remained significant after controlling for age, gender and duration of dialysis. However, all the PTH values among osteoporotic patient in the three BMD studies were higher than normal and osteopenic patients. The current study found that increased duration of dialysis is associated with lower BMD, p<0.05,. These findings correlate with a previous study conducted in Japan that suggested a strong association with the hyperparathyroidism because of long duration on dialysis therapy and generalized bone loss [27]. This is attributed to a combination of factors, including reduction in vitamin D synthesis, hyperphosphatemia, hypocalcemia, and PTH skeletal resistance which will eventually increase the risk of mortality and morbidity as a result of bone disease and fractures [11]. The hyper activated PTH is considered to deteriorate bone mechanical properties, to rescue the state of hypocalcemia among ESRD patients, on the expense of changing bone structure and reducing bone mass [28].

The percentage of patients with osteopenia and osteoporosis were significantly higher among patients ≥60 years of age relative to patients <60 years of age for LS and Hip BMD. This goes against the findings of a study that reported positive correlation between the Spine BMD and age [29] but it correlates with the findings of previous studies [9, 30]. In contrast to other study [9] this research found a significant association between BMD and calcium level (P<0.03) and calcium supplementation (P<0.05 at spine BMD). This might be due to the different patterns of supplemental calcium ingestion among the patients and not due to the pathophysiological problems related to the change in serum PTH given the fact that high serum PTH should be accompanied with low serum calcium as lower ionized calcium levels may influence protein-RNA interactions at the 3-untranslated region of PTH mRNA leading to increased PTH mRNA stability [31].

In this study, eight of the sixteen renal transplant patients had osteoporosis and four had osteopenia. Even though no significant association was found between BMD and history of renal transplantation according to LS or hip (P = 0.059, P = 0.718 respectively), the relationship between bone loss and long term use of immunosuppressive agents in transplant patients requires further study. In a another study, patients who had received glucocorticoids had lower BMD Z-score in both femoral neck and spine compared with patients who had received no such treatment [25]. These patients should thus be considered candidates for a closer continuous monitoring.

The relationship between albumin and BMD has rarely been reported. In a study by Lai et al. showed that there was no association between the serum albumin level and BMD in the hemodialysis patients [32]. Meanwhile, another study revealed that the patients with an albumin level above 4.1 g/dl have a higher BMD [9]. Likewise, this study showed a significant correlation between serum albumin, hip and spine BMD (P<0.05) as osteoporotic patients had a lower serum levels of albumin in the three occasions and this was statistically significant in the LS and Hip, this remained the case even after controlling for Age, gender and duration of dialysis (P = 0.025, P = 0.010 in the LS and hip, respectively)

### Strengths and limitations

This study included relatively a large number of patients undergoing hemodialysis at NNUH. It is the only center providing peritoneal dialysis therapy and the number of hemodialysis patients in this unit represents about 20% of all hemodialysis patients in the West Bank, Palestine. So, the demographic, clinical and biochemical characteristics of included hemodialysis patients are likely to be generalizable to hemodialysis population in Palestine. Up to author’s knowledge, it is the first study of its style in Palestine that focuses on this special category of population. The results can be used as framework for further future studies for either follow up or improving patients’ quality of life by closely monitoring and paying more attention for the patients at high risk according to the clinical, demographic and biochemical predictive measures. Our study has some limitations that should be taken into consideration when interpreting the study results. Given the cross-sectional design of the study, we were unable to assess changes in BMD and bone turnover markers over time. BMD sites were limited to the hip and LS and did not include the radius. Other limitation of this study is the small correlation coefficient values observed that might be due to the diverse population in this study; a wide variety of ages, all genders, history of transplantation, etc. Lastly, the small number of PD patients included in this study could have limited its ability to show significant relation between dialysis type and MBD.

## Conclusion

This study showed that Palestinian patients with ESRD have low BMD at the hip and spine and the prevalence of osteoporosis and osteopenia is high. This study also adds further evidence that high serum PTH levels is associated with low BMD and makes the patients at increased risk of osteoporosis and bone fractures. Therefore, those patients should be closely monitored especially those with more than one risk factor such as female patients with longer duration on dialysis. They should be advised to stick to their medications to adjust the serum level of PTH and the other minerals to decrease the risk of fractures. Moreover, more attention should be paid for these category of patients to decrease the incidence of falling down and the resulting fractures that might lead to mortality and morbidity.

## Supporting information

S1 Data (SAV)

## Abbreviations

ALP: Alkaline phosphatase
BMD: Bone mineral density
CKD: chronic kidney disease
DEXA: dual-energy X-Ray absorptiometry
ESRD: End stage renal disease
GFR: Glomerular filtration rate
HD: Hemodialysis
LS: Lumbar spine
NNUH: Najah national university hospital
PMP: per million population
PTH: parathyroid hormone
WHO: world health organization
